# Supplementary material for: A Systematic Review and Meta-Analysis of Advanced Biomarkers for Predicting Incident Cardiovascular Disease among Asymptomatic Middle-Aged Adults
Source: Int J Mol Sci. 2022 Nov 4;23(21):13540. doi: 10.3390/ijms232113540 (PMC9656299; doi:10.3390/ijms232113540)
Supplement: Supplementary file 1 [file ijms-23-13540-s001.zip › ijms-1958683-supplementary.pdf]

Supplementary material

Supplementary Table S1. Search strategy using MeSH Terms.

MeSH Terms
("metabolomics"[All Fields] OR "fibrinogen"[All Fields] OR “homocysteine” [All Fields] OR “hs-CRP” [All Fields] OR “C-reactive protein” [All Fields] OR “IL-6” [All Fields] OR “ApoB/ApoA” [All Fields] OR “lipoproteinA” [All Fields] OR "apolipoproteins"[All Fields] OR “Lipoprotein-associated phospholipase A2” [All Fields] OR “secretory phospholipase A2” [All Fields] OR “LDL-P” [All Fields] OR “Oxidized LDL” [All Fields] OR “myeloperoxidase” [All Fields]) AND (“screening”[All Fields] OR “prediction”[All Fields] OR “predict”[All Fields]) AND ("cardiovascular diseases"[All Fields] OR “cardiovascular disease” [All Fields] OR “heart disease”[All Fields] OR mortality [All Fields]) AND English[lang] AND ("adult"[MeSH Terms] OR "middle aged"[MeSH Terms]) AND hasabstract[text] AND "2009/02/01"[PDat] : "2019/11/30"[PDat] AND "humans"[MeSH Terms] AND English[lang]).

Supplementary Table S2. Summary of all papers included.

Name of article	Type of study	Quality of study	Authors	Journal	Year	Biomarkers	Population	Main Findings
The association between circulating endostatin levels and incident myocardial infarction	Nested case-control study	Good	Toralph Ruge, et al.	Scandinavian Cardiovascular Journal	2019	Endostatin	All cases with acute MI in the age group 25-64 years from prospectives MONICA, VIP and MSP cohorts in Sweden were selected (n=533), matched with two referents per case for sex, age, cohort, date of health examiation and geographical area (n=1003).	Higher endostatin levels was associated with an increased incidence of MI when comparing cases and their age-, sex and cohort-matched controls (OR 1.5, IC 95% 1.32-1.71, p <0.01), but this association was attenuated when adjusted for stablished CV risk factors (OR 1.16, IC 95% 1.01-1.34, p 0.04) and abolished when adjusted for CRP also (OR 1.11, IC 95% 0.95-1.28, p 0.18). The association between endostatin and MI was predominantly seen in women.
Astronaut Cardiovascular Health and Risk Modification (Astro-CHARM) Coronary Calcium Atherosclerotic Cardiovascular Disease Risk Calculator	Cohort (Prospective study)	Good	Amith Khera, et al.	Circulation	2018	hsCRP	Participants were middle-age individuals (40-65 years) pooled from study participants from examination 1 of MESA, phase 1 of the Dallas Heart Study (DHS), and the PACC study (Prospective Army Coronary Calcium Project) for the derivation cohort (n=7382).	Over a median of 10.9 years, 304 hard ASCVD events occurred. Associations of risk factors with study outcomes were assessed by using Cox proportional hazards models. hsCRP showed higher risk to the endpoint (composite of nonfatal MI, nonfatal stroke, or death from CHD or stroke). (HR per 1 SD unit (4.8) 1.1 (CI 95% 1.0-1.2, p 0.009).
Prognostic value of suPAR and hs-CRP on cardiovascular disease	Cohort (Prospective study)	Good	Marie Zöga Diederichsen et al.	Atherosclerosis	2018	soluble urokinase plasminogen activator receptor (suPAR), hsCRP	1179 men and women aged 60 and 50 years from DanRisk study	Follow-up was 6.5 years and 73 events occurred from baseline to follow-up. In Cox analyses adjusted for traditional risk factors and CAC score, both suPAR (HR 1.20, CI 95% 1.04-1.39) and hsCRP (HR 1.03, CI 95% 1.003-1.05) were associated with CV event. Stratification for age showed that suPAR and CRP were associated with CV events among 60 years old subjects. Stratification for gender showed that suPAR was associated among women while CRP was associated with events among both men and women.
Impact of systemic inflammation on the relationship between insulin resistance and all-cause and cancer-related mortality	Cohort (Prospective study)	Good	Da Young Lee, et al.	Metabolism Clinical and Experimental	2018	HOMA-IR, hsCRP	165,849 subjects aged 20 years or older, who participated in the health-screening programs at the Kangbuk Samsung Hospital Total Healthcare Center (or its clinics) in Seoul and Suwon, South Korea. Mean age 39.5 (SD 9.2)	The mean follow-up period was 8.54 ± 1.42 years, with a total of 1316 deaths (182 from CVD) ocurred. Subjects in Q4 of HOMA-IR (2.31-32.7) had after adjusted for clasical risk factors and hsCRP, TC, HDL and TG, HR for all-cause mortality 1.20 (CI 95% 1.01-1.43), CV mortality 0.75 (0.47-1.19) and cancer-related mortality 1.40 (1.1-1.78). Subjects in Q4 of hsCRP (≥ 1.1 mg/L) had after adjusted same above but with HOMA-IR, HR for all-cause mortality 1.40 (CI 95% 1.18-1.66), CV mortality 1.58 (0.96-2.58) and cancer related mortality 1.59 (1.14-1.88), and p for trend between quartiles were significants in all three outcomes.

A Multimodality Strategy for Cardiovascular Risk Assessment: Performance in Two Population-Based Cohorts	Cohort (Prospective study)	Good	James A. de Lemos, et al.	Circulation	2018	hsTn, hsCRP, NT-proBNP	6621 participants from MESA study 45-84 years old (these participants were excluded for the current analysis) and 2202 participants from DHS study aged 30-65.	In the DHS, over a median follow-up period of 10.3 years, 179 global CVD events occurred, including 96 ASCVD events. NT-ProBNP in continuous analysis and categorical analysis (≥100pg/mL) after multivariable adjustment for risk factors had HR 1.19 (CI 95% 1.01-1.41) and 1.88 (1.29-2.75) for CV endpoint. The same hs-cTnT in continuos analysis and categorical analysis (≥5ng/L) after multivariable adjustment had HR 1.17 (1.01-1.35) and 1.46 (1.01-2.11) for CV endpoint. But hs-CRP in both continuous and categorical analysis (≥3 mg/L) didn’s have significant HR for CV endpoint 0.97 (0.82-1.15) and 1.06 (0.78-1.46).
Twenty-Year Predictors of Peripheral Arterial Disease Compared With Coronary Heart Disease in the Scottish Heart Health Extended Cohort (SHHEC)	Cohort (Prospective study)	Good	Hugh Tunstall-Pedoe, et al.	Journal American Heart Association (AHA)	2017	hsCRP, Fibrinogen, homocysteine, hsTnI, NTproBNP, Lipoprotein (a), GGT, Uric acid, cistatinC	15,737 participants from Scottish Heart Health Extended Cohort (SHHEC) with mean age of 49 (SD 8.3)	After adjustment for ASSIGN risk factors, some biomarkers showed significant HR for CHD. hsCRP 1.22 (1.15-1.29). Fibrinogen 1.09 (1.05-1.12). Homocysteine 1.08 (1.05-1.12). hsTnI 1.29 (1.14-1.53). NT pro-BNP 1.21 (1.16-1.27). GGT 1.07 (1.02-1.13). Uric acid 1.04 (1-1.09). Cystatin-C 1.13 (1.10-1.16). But others didn’t have significant differences such as C-peptide 1.02 (0.97-1.06), lipoprotein (a) 1.01 (0.97-1.05) or Ferritin 1.02 (0.97-1.07).
Pentraxin 3, ficolin-2 and lectin pathway associated serine protease MASP-3 as early predictors of myocardial infarction - the HUNT2 study	Case-control study	Good	Inga Thorsen Vengen, et al.	Scientific Reports-Nature	2017	Pentraxin-3, ficolin1,2 and 3, MASP-3, MAS-1	735 subjects (366 cases IM and 369 control matched age and gender) from HUNT2 study were selected for the present case-control study with mean age 53 years old (range 29-62)	Highest tertile of pentraxin-3, ficolin-2 and MASP-3 was associated with an increased incidence of MI after adjustments for traditional risk factors 2.79 (CI 95% 1.83-4.24, p 0.001), 1.55 (1.04-2.30) and 0.63 (0.43-0.94) respectively. Results from logistic regression including these three biomarkers showed that two highest tertiles of pentraxin-3 and highest tertile of MASP-3 remained significant after adjustments for traditional risk factors 1.61 (CI 95% 1.07-2.44, p 0.023), 2.90 (1.89-4.47, p 0.0005) and 0.52 (0.34-0.79, p 0.002) respectively.
Circulating Ceramides Predict Cardiovascular Outcomes in the Population-Based FINRISK 2002 Cohort	Cohort (Prospective study)	Good	Aki S. Havulinna, et al.	Arteriosclerosis, Thormobosis Vascular Biology-AHA	2016	Ceramides	8101 participants from the FINRISK 2002 general population cohort were included in this study with a median age of 48.5 years old. For the current analysis only 7,705 individuals without MACE prior to baseline were analysed.	Ceramide (d18:1/16:0), ceramide(d18:1/18:0) and ceramide (d18:1/24:1) showed association with incident MACE in the Cox regression analyses 1.14 (CI 95% 1.04-1.25), 1.21 (1.11-1.33) and 1.14 (1.04-1.26) respectively after adjustment for traditional risk factors but not in the fatal incident MACE after adjustments. These association remained unchanged after additional adjustment for CRP.
Plasma and blood viscosity in the prediction of	Cohort (Prospective study)	Good	Sanne AE Peters, et al.	European Journal of Preventive Cardiology	2016	Plasma and blood viscosity	3,386 individuals from Scottish Heart Health Extended Cohort (SHHEC) with mean age of 49 (SD 11).	Plasma viscosity showed a significant association with cardiovascular disease and total mortality, including after adjustment for traditional CV risk factors and

cardiovascular disease and mortality in the Scottish Heart Health Extended Cohort Study								fibrinogen (close related with level of plasma viscosity) HR 1.09 (CI 95% 1.01-1.18, p 0.03) and 1.13 (CI 95% 1.04-1.22, p 0.003) respectively, but not whole blood viscosity, corrected blood viscosity, relative blood viscosity or haematocrit showed significant differences. Categorical analysis showed the same results for the highest tertile of plasma viscosity, with HRs 1.28 (CI 95% 1.13-1.45) and 1.45 (1.25-1.68).
Beyond Coronary Calcification, Family History, and C-reactive Protein: Cholesterol Efflux Capacity and Cardiovascular Risk Prediction	Cohort (Prospective study)	Good	Purav Mody, et al.	JACC	2016	Cholesterol Efflux Capacity	1,972 participants from DHS cohort study 30 to 65 years of age were selected to the final analysis of cardiovascular outcomes	Cholesterol efflux capacity (CEC) was assessed by measuring the efflux of fluorescent-labeled cholesterol from J774 macrophages to apoliprotein B depleted plasma. Among the 1,972 participants included in analysis, 97 had a first ASCVD event (28 MIs, 32 strokes, 5 coronary artery bypass graft surgeries, 11 percutaneous coronary interventions and 21 cardiovascular deaths) over a median follow up of 9.4 years (95% CI 9.0, 9.8). In a Cox proportional hazards model for incident ASCVD with traditional risk factors CEC showed inversily association with incident ASCVD 0.35 (CI 95% 0.23-0.55).
Inverse linear associations between liver aminotransferases and incident cardiovascular disease risk: The PREVEND study	Cohort (Prospective study)	Good	Setor K. Kunutsor, et al.	Atherosclerosis	2015	AST, ALT	6899 participants from PREVEND study with mean age of 48 (SD 12)	During a median follow-up of 10.5 years, 729 incident CVD events were recorded. An analysis adjusted for the established classic risk factors, other covariables such as BMI, alcohol consumption, etc and further adjustment for loge hsCRP, the HRs for CVD and ALT was 0.88 (CI 95% 0.80 to 0.96, p 0.003) and AST was 0.92 (CI 95% 0.84-0.99, p 0.029). In separate analyses for CHD and stroke, ALT remained a significant association after adjustment for CHD, but not a significant association for stroke, while AST had not a significant association for CHD neither stroke.
Serum Alkaline Phosphatase and Risk of Incident Cardiovascular Disease: Interrelationship with High Sensitivity CReactive Protein	Cohort (Prospective study)	Good	Setor K. Kunutsor, et al.	PLOS One	2015	Alkaline Fosfatase, hsCRP	6,974 participants from PREVEND cohort were included in the current study with mean age of 48 years old	During a median follow-up of 10.5 years, 737 incident CVD events were recorded. An analysis adjusted for established risk factors, other covariables such as BMI, alcohol consumption, etc and further adjustment for loge hsCRP, the HRs for CVD and highest quintile ALP compared with the others was 1.24 (CI 95% 1.05 to 1.45, p 0.009), but was not significant in a separate analysis for CHD or stroke. Association of hsCRP with incident CVD was showed, being after the same adjustment described above HR 1.26 (CI 95% 1.17 to 1.38, p <0.001), and in separate analyses for CHD and

								stroke it remained with a significant association for each one.
Circulating gamma glutamyltransferase and prediction of cardiovascular disease	Cohort (Prospective study)	Good	Setor K. Kunutsor, et al.	Atherosclerosis	2014	Gamma glutamyltransferase	6969 subjects with non-missing information on GGT levels and several CVD risk markers from PREVEND cohort with a mean age of 48 (SD 12) years old were included	During a median follow-up of 10.5 years, 735 incident CVD events were recorded. In an analysis after adjustment for established risk factors, other potential cofounders and CRP, the HR per 1 standard change in GGT was 1.18 (CI 95% 1.06 to 1.30, p 0.002). In a separate analysis for stroke after the same adjustment remained significant, but not for CHD. In a subgroup analysis for age, significant differences were displayed independent of the age, but in younger subjects than 50 years old was strongest.
Sleep duration, C-reactive protein and risk of incident coronary heart disease e results from the Framingham Offspring Study	Cohort (Prospective study)	Good	J. Liu, et al.	Nutrition, Metabolism & Cardiovascular Diseases	2013	CRP	3,381 individuals from FOS cohort were included in the current analysis	Over the 20 years follow-up, a total of 491 newly developed incident CHD were recorded. The HRs of incident CHD for those with CRP levels of 1-3 mg/L and ≥3 mg/L compared to those with CRP levels <1mg/L after adjusting for age, gender and traditional risk factors for CHD were 1.05 (0.83, 1.32, p < 0.70), and 1.28 (0.99, 1.65, p < 0.06), respectively.
N-terminal pro-B-type natriuretic peptide and the prediction of primary cardiovascular events: results from 15-year follow-up of WOSCOPS	Cohort (Prospective study)	Good	Paul Welsh, et al.	European Heart Journal	2012	NT-ProBNP	4128 moderately hypercholesterolaemic men included in the clean CVD cohort from WOSCOPS clinical trial have been included in the current analysis. Clean CVD cohort: patients with positive Rose angina, stroke/TIA, ECG abnormalities, claudication and history of another type of vascular disease were excluded	Over a median of 14.7 years of follow-up, 1357 CVD events were recorded. After an adjustment for randomized treatment, age, and others cardiovascular risk factors such as BMI, smoking, diabetes, etc. plus CRP, HRs 1 SD increase in log NT-proBNP for all CVD events was 1.20 (CI 95% 1.13-1.27, p <0.001), but was not significant for CHD events after adjusts. However, when the fatal events were analyzed both CVD deaths and CHD deaths had significant differences after the adjustments, HRs 1.29 (1.11-1.48, p 0.001) and 1.22 (1.03-1.45, p 0.02) respectively.
Use of Serum Homocysteine to Predict Cardiovascular Disease in Korean Men with or without Metabolic Syndrome	Case-control study	Poor	Ji Yeon Kang, et al.	Journal of Korean Medical Science	2012	Homocysteine	Retrospective case-control study with 428 subjects.The subjects were assigned in about 2:1 ratio to either the non-CVD group or CVD group. A total of 138 CVD subjects and 290 patients matched for age and the number of MetS components. In addition, non-CVD and CVD groups were divided in four subgroups depending on the presence of MetS.	An analysis of covariance after adjustment for age (ANCOVA) was carried out to assess the relationship between homocysteine and differents four groups of subjects and significant differences were found, being the levels of homocysteine in normal non-CVD subjects of 10.89 +/- 2.86, in MetS non-CVD subjects 13.11 +/- 3.99, CVD subjects 13.02 +/- 3.6 and finally in MetS CVD subjects 14.60 +/- 2.92, p= 0.001. In addition, a stepwise regression analysis was carried out to assess whether homocysteine predicts CVD in MetS and non-MetS subject, being significant in both.

Exploring clinical predictors of cardiovascular disease in a central Australian Aboriginal cohort	Cohort (Prospective study)	Fair	Joanne N Luke, et al.	European Journal Preventive Cardiology	2012	total/HDL cholesterol ratio, non-HDL cholesterol, GGT, Uric acid, Fasting insulin, Albumin/Cr ratio, fasting glucose, 2 h glucose, fibrinogen	739 subjects from three central Australian Aboriginal communities were included in the study with a mean age of 35 ±16 years old. For the current analysis, only 625 subjects younger than 65 years old and free of prior CVD were included.	During the 10-year follow-up period sixty-eight participants experienced an incident CVD event. For many biomarkers the odds ratio (95% CI) for CVD were calculated using cut-points. Total: HDL Cholesterol ≥5.7 OR 3.78 (1.88-7.59). Non-HDL cholesterol ≥4.3 mmol/L, OR 3.23 (1.84, 5.66). HDL Cholesterol ≤0.9 mmol/L OR 2.05 (1.04, 4.02). Gamma-glutamyl transferase ≥70 U/L, OR 2.66 (1.56, 4.55). Uric Acid ≥350 mmol/L, OR 1.54 (0.86, 2.76). Fasting insulin ≥ 20μU/mL, OR 1.74 (1.02, 2.97). Albumin:Creatinine ≥5.7 mg/mmol, OR 2.65 (1.54, 4.53). Fasting glucose ≥4.8 mmol/L, OR 4.10 (2.37, 7.10). 2 hour glucose ≥6.0 mmol/L, OR 1.93 (1.13, 3.30). Fibrinogen ≥3.5 g/L,1.62 (0.95, 2.78).
Free Protein S Level as a Risk Factor for Coronary Heart Disease and Stroke in a Prospective Cohort Study of Healthy United Kingdom Men	Cohort (Prospective study)	Good	Gie Ken-Dror, et al.	American Journal of Epidemiology	2011	Protein S	3.052 middle-aged (49-64 years) men were included from NPHS-II cohort with a mean age of 56.1 years (SD 3.48), and a median of follow-up time of 13.7 years.	During the follow-up period, 297 subjects (9.7%) experienced a CHD event and 98 subjects (3.2%) experienced a stroke event. The association betwwen free protein S and CHD was studied dividing free protein S in five knots. After an adjustment for age, BMI, diabetes, clinic, smoking, HDLc, TC, SBP, and use of lipid or blood pressure lowering medications the HRs estimate for CHD for the fifth knots compare with the first knot was 1.84 (95% CI 1.11, 3.06; p 0.018).
Systemic chemokine levels, coronary heart disease, and ischemic stroke events. The PRIME Study	Nested case-control study	Good	F. Canouï-Poitrine, et al.	Neurology	2011	RANTES/CCL-5, IP-10/CXCL10, MCP-1 (CCL-2), eotaxin-1 (CCL11)	9,711 subjects from WHO MONICA cohort had a 10 year follow-up period. At the end of 10 years of follow-up 635 men developed a first coronary event and 98 a first ischemic stroke but baseline plasma samples were avaible respectively for 621 and 95 men. 1,242 and 190 matched controls (2:1 controls per case) were selected from the initial cohort and used for analysis (nested case-control study design).	Hazard ratios (HR) and 95% confidence intervals (CIs) of each chemokine were estimated for CHD and for ischemic stroke. In unadjusted analysis, higher RANTES (HR 1.17; 95% CI 1.02–1.34; p 0.03) and eotaxin-1 levels (HR 1.16; 95% CI 1.03–1.32; p 0.014) were associated with CHD whereas IP-10 (HR 0.96; 95% CI 0.87–1.06; p 0.44) and MCP-1 (HR 0.98; 95% CI 0.77–1.46; p 0.73) were not. However, after adjustment for traditional risk factors and medications, hs-CRP and fibrinogen, RANTES (HR 1.10; 95% CI 0.95–1.29; p 0.22) and eotaxin-1 (HR 1.09; 95% CI 0.95–1.24; p 0.20) were no longer associated with CHD. In unadjusted analyses, higher RANTES (HR 1.56; 95% CI 1.04 –2.34; p 0.032), IP-10 (HR 1.48; 95% CI 1.06 –2.08; p 0.022), and eotaxin-1 (HR 1.53; 95% CI 1.05–2.24; p 0.026) were associated with ischemic stroke. These associations were unaffected by further adjustment as above. MCP-1, however, was not associated with ischemic stroke even in unadjusted analysis. When RANTES, IP-10, and eotaxin-1 were considered in the same model adjusted RANTES (HR 1.83; 95% CI 1.21–2.75; p 0.004), and IP-

								10 (HR 1.95; 95% CI 1.33–2.87; p 0.001) but not eotaxin-1 (HR 1.14; 95% CI 0.76 –1.71; p 0.54) remained associated with ischemic stroke.
Oral postmenopausal hormone therapy, C-reactive protein and cardiovascular outcomes	Cohort (Prospective study)	Good	Emily G. Kurtz, et al.	Menopause	2011	hsCRP	26,791 participants from Women's Health Study (WHS) were included in the current study with a median age of 52.9 years old. Participants were followed for a mean of 10 years for the occurrence of a first major cardiovascular event (CVE).	The total cohort was divided into hormone non-users and hormone users. Firstly in a continuous analysis the RR for lnCRP was 1.27 (95% CI, 1.13 to 1.44) for HT non-users and 1.22 (95% CI, 1.07 to 1.40) for HT users. In addition, relative risk was calculated according to quintile of CRP based on HT non users and HT users levels and categories defined by AHA/CDC (<1mg/L, ≥1-<3 mg/L and ≥ 3 mg/L). After risk factor-adjusted RR highest quintile, based on HT non-users levels (>4.18 mg/L), significantly predicted CVE in HT non-users RR: 2.85 (95% CI, 1.62 to 5.00), but not in HT users. However, based on HT users levels (>6.44 mg/L), highest quintile predicted CVE RR: 1.88 (95% CI, 1.14 to 3.11). In a fit model in wich non-users with CRP< 1 mg/L were the reference group after adjusting HT users with CRP ≥3 had a RR of 1.93 (1.38–2.69) while non-users had a RR of 1.92 (1.35–2.72).
Circulating soluble urokinase plasminogen activator receptor predicts cancer, cardiovascular disease, diabetes and mortality in the general population	Cohort (Prospective study)	Fair	J. Eugen-Olsen, et al.	Journal of Internal Medicine	2010	SuPAR, hsCRP	2,602 participants from MONICA cohort with validated suPAR levels were selected for the current study. Participants were 41,51,61 and 71 years old at baseline of the study (subanalysis were carried out) and were followed for a median of 12.6 years (range 0.17-13.6)	Differents four endpoint were studied: cancer, CVD, T2DM and death. During the follow-up period 301 incident cases of CVD were recorded. Subanalysis for age were realized. After adjustment for variables included in Frammingham risk score and CRP or suPAR depending of biomarkers analysed, HRs for suPAR and CVD in 41 years old subjects was 1.32 (1.14–1.52) p <0.001, in 51 years old subjects was 1.22(1.03–1.44) p 0.02 and in 61 years old subjects 1.09(0.95–1.26) p 0.23, and HRs for CRP>3mg/L and CVD in 41 years old subjects was 1.24 (0.51–3) p 0.63, in 51 years old subjects was 1.78 (0.92–3.45) p 0.09 and in 61 years old subjects was 2.05 (1.1-3.86) p 0.02. The data from subjects 71 years old are not displayed here because it is out our objetive. For mortality and suPAR HRs were all significant after adjustment in 41,51,61 and 71 years old subjects, but for CRP>3 mg/L only in 61 years old subjects.
Low levels of IgM antibodies to phosphorylcholine predict cardiovascular	Nested case-control study	Good	Ulf de Faire, et al.	Journal of Autoimmunity	2010	IgM anti phosphorilcholine	In 4232 subjects (2039 men and 2193 women), 211 incident cases of first CVD events (77 with MI, 85 with angina pectoris and 49 with ischemic stroke). A nested case-control study was carried	Association between IgM antibodies PC and risk of CVD was assessed, showed not significant association after adjustment for cardiovascular risk factors for those whitin in lowest quartile (values below 29.7 U/l) compare with those in highest quartile (values above

disease in 60-year old men: Effects on uptake of oxidized LDL in macrophages as a potential mechanism							out and for each case three controls were randomly selected, matched for gender and age. Finally, 633 controls and 211 case were included in the current study.	72.73 U/l). However, when subanalyses were carried out by gender, this association was stronger and significant after adjustment in men RR 1.96 (95% CI 1.09 3.55) p 0.025, but not in women RR 0.77 (0.35-1.71) p 0.525.
Does fibrinogen add to prediction of cardiovascular disease? Results from the Scottish Heart Health Extended Cohort Study	Cohort (Prospective study)	Good	Mark Woodward, et al.	British Journal of Haematology	2009	Fibrinogen	12,352 subjects (6114 men and 6238 women) from Scottish Heart Health Extended Cohort and Scottish MONICA study without missing values for any of the ASSIGN or Framingham variables were included for the current analyse	During follow-up (10-21 years, median 19.2 years), 2448 (1508 men and 940 women) had a CVD event. After adjustment for age, framingham and ASSIGN variables an increase of one unit (g/l) in fibrinogen was associated with an increase in cardiovascular disease, with HRs for CVD of 1.09 (95% CI 1.02-1.19) for men and 1.09 (95% CI 1.01-1.18) for women. Similar estimates were found for CVD death, but data is not shown.
A consultation-based method is equal to SCORE and an extensive laboratory-based method in predicting risk of future cardiovascular disease	Cohort (Prospective study)	Good	Ulla Peterson, et al.	European Journal of Cardiovascular Prevention and Rehabilitation	2009	LDL/HDL ratio, hsCRP, insulin-like growth factor-I (IGF-1) and symmetric dimethyl arginine (SDMA)	689 participants from the baseline of Söderåkra Cardiovascular Risk Factor study after exclusion of participants with a history of prevalent CVD.	During the follow-up period for 17 years, 69 participants died and the first fatal or nonfatal event of CVD occurred in 71 participants. HRs with 95% CIs using univariate Cox's proportional hazard regression analyses for first major nonfatal or fatal cardiovascular event were calculated after adjustement for the others variables included in the differents models such as hypertensión, age, gender, smoking, family history CVD, treatment for diabetes and hypertension, SBP and waist/height ratio. HR for LDL/HDL ratio was 1.6 (1.4–2.0) p <0.001. HR for hsCRP was 1.4 (1.1–1.8) p 0.010. HR for IGF-1 was 0.35 (0.14–0.87) p 0.024. HR for SDMA was 7.4 (1.0–54) p 0.048.
New risk markers may change the HeartScore risk classification significantly in one-fifth of the population	Cohort (Prospective study)	Fair	MH Olsen, et al.	Journal of Human Hypertension	2009	hs-CRP, NT-ProBNP, urine albumin/ creatinine ratio	1,988 healthy subjects were included after exclusion of 472 subjects from the baseline study with known diabetes, prior myocardial infarction or stroke. These healthy subjects were classied using HeartScore in high risk (559) and in low-moderate risk (1429) depending expected 10-year risk of CV death above or below 5%.	In univarate Cox-regression analyses for hsCRP, HRs for composite of CV end-point and CV death in low-moderate risk subjects was 1.9 (1.0-3.5) p<0.05 and 1.1(0.4-3.2) respectively and in high risk subjects was 1.9 (1.2-3.1) p<0.05 and 2.5 (1.4-4.7) p<0.01 respectively, for NT-ProBNP HRs for CVD and CV death in low-moderate risk subjects was 1.1 (0.6-2.1) and 2.1(0.6-6.9) not significants and in high risk subjects was 2.6 (1.6-4.3) p<0.001 and 4.7 (2.5-9.1) p<0.001 respectively, and finally for UACR HRs for CVD and CV death in low-moderate risk subjects was 2.1 (1.3-3.5) p<0.01 and 3.2 (1.6-6.4) p<0.01 respectively and in high risk subjects was 2.1 (1.5-3.0) p 0.001 and 2.5 (1.6-3.7) p<0.001.

Supplementary Table S3. Studies assessing fibrinogen.

Citation	Type of study	Quality of study	Population	CV outcomes	Length of follow-up	Main results
Hugh Tunstall-Pedoe, et al. Journal American Heart Association (AHA). 2017. doi: 10.1161/JAHA.117.005967 PMID: 28923990; PMCID: PMC5634266.	Cohort (Prospective study)	Good	15,737 participants from Scottish Heart Health Extended Cohort (SHHEC) with mean age of 49 (standard deviation (SD) 8.3).	Coronary heart disease (CHD) was defined as ICD 9[a] codes 410 to 414 and ICD 10 I20 to I25 while peripheral artery disease (PAD) was defined as ICD 9 440.2, 443.9, 250.6 and ICD 10 I70.2, I73.9, E10.5, E11.5, E12.5, E13.5, E14.5.	19.9 years was mean follow-up	3098 CHD events and 499 PAD events occurred. After adjustment Fibrinogen showed a hazard ratio (HR) 1.09 (confidence interval (CI) 95% 1.05-1.12).
Joanne N Luke, et al. European Journal Preventive Cardiology. 2012. doi: 10.1177/2047487312437713. PMID: 22345691.	Cohort (Prospective study)	Fair	739 subjects from three central Australian Aboriginal communities were included in the study with a mean age of 35 ±16 years old. For the current analysis, only 625 subjects younger than 65 years old and free of prior CVD were included.	The composite of cardiovascular (CV) events basis in ICD-9 and ICD-10 codes included coronary heart disease (ischaemic heart disease, acute myocardial infarction or angina pectoris), stroke (ischaemic, haemorrhagic or unspecified), PAD or chronic heart failure	10-year follow-up period	During follow-up period sixty-eight participants experienced an incident cardiovascular diseases (CVD) event. Fibrinogen showed for CVD in categorical analysis with a cut-points ≥3.5 g/L[b], odds ratio (OR) was 1.62 (CI 95% 0.95-2.78).
Mark Woodward, et al. British Journal of Haematology. 2009. doi: 10.1111/j.1365-2141.2009.07778.x. PMID: 19549268.	Cohort (Prospective study)	Good	12,352 subjects (6114 men and 6238 women) from Scottish Heart Health Extended Cohort and Scottish MONICA study without missing values for any of the ASSIGN or Framingham variables were included for the current analyse	CVD endpoints were basis in ICD9 and ICD10 including deaths attributed to a cardiovascular cause, any hospital discharge diagnosis postrecruitment of coronary heart disease, or cerebrovascular disease, r surgical codes for coronary artery bypass graft or percutaneous coronary Angioplasty.	During follow-up (10-21 years, median 19.2 years)	2448 (1508 men and 940 women) had a CVD event. After adjustment an increase of one unit (g/L) in fibrinogen was associated with an increase in cardiovascular disease, with HRs for CVD of 1.09 (95% CI 1.02-1.19) for men and 1.09 (95% CI 1.01-1.18) for women.

[a] international classification disease (ICD-9), [b] grams/liter (g/L)

Supplementary Table S4. Studies assessing gamma glutamyl-transferase (GGT).

Citation	Type of study	Quality of study	Population	CV outcomes	Length of follow-up	Main results
Hugh Tunstall-Pedoe, et al. Journal American Heart Association (AHA). 2017. doi: 10.1161/ JAHA.117.005967 PMID: 28923990; PMCID: PMC5634266.	Cohort (Prospective study)	Good	15,737 participants from Scottish Heart Health Extended Cohort (SHHEC) with mean age of 49 (standard deviation (SD) 8.3).	Coronary heart disease (CHD) was defined as international classification diseases (ICD-9) codes 410 to 414 and ICD 10 I20 to I25 while peripheral artery disease (PAD) was defined as ICD 9 440.2, 443.9, 250.6 and ICD 10 I70.2, I73.9, E10.5, E11.5, E12.5, E13.5, E14.5.	19.9 years was mean follow-up	3098 CHD events and 499 PAD events occurred. After adjustment for ASSIGN risk factors GGT showed a hazard ratio (HR) 1.07 (confidence interval (CI) 95% 1.02-1.13).
Setor K. Kunutsor, et al. Atherosclerosis. 2014. doi: 10.1016/j.atherosclerosis. 2014.12.045. Epub 2014 Dec 23. PMID: 25555268.	Cohort (Prospective study)	Good	6969 subjects with non-missing information on GGT levels and several cardiovascular diseases (CVD) risk markers from PREVEND cohort with a mean age of 48 (SD 12) years old were included	Acute myocardial infarction, acute and subacute ischaemic heart disease, coronary artery bypass grafting or percutaneous transluminal coronary angioplasty, subarachnoid haemorrhage, intracerebral haemorrhage, other intracranial haemorrhage, occlusion or stenosis of the precerebral or cerebral arteries, other vascular interventions such as percutaneous transluminal angioplasty or bypass grafting of aorta and peripheral vessels, and mortality.	A median follow-up of 10.5 years	735 incident CVD events were recorded. In an analysis after adjustment for established risk factors, other potential cofounders and C-reactive protein (CRP), the HR per 1 standard change in GGT was 1.18 (CI 95% 1.06 to 1.30, p 0.002). In a separate analysis for stroke after the same adjustment remained significant, but not for CHD. In a subgroup analysis for age, significant differences were displayed independent of the age, but in younger subjects than 50 years old was strongest.
Joanne N Luke, et al. European Journal Preventive Cardiology. 2012. doi: 10.1177/ 2047487312437713. PMID: 22345691.	Cohort (Prospective study)	Fair	739 subjects from three central Australian Aboriginal communities were included with a mean age of 35 ±16 years old. 625 subjects younger than 65 years old free of prior CVD were included.	The composite of CV events basis in ICD-9 and ICD-10 codes included coronary heart disease (ischaemic heart disease, acute myocardial infarction or angina pectoris), stroke, PAD or chronic heart failure.	10-year follow-up period	During follow-up period sixty-eight participants experienced an incident CVD event. GGTshowed for CVD in categorical analysis with a cut-points ≥70 international units/liters (UI/L) OR was 2.66 (CI 95% 1.56-4.55).

PRISMA 2020 Checklist

Section and Topic	Item #	Checklist item	Location where item is reported
**TITLE**			
Title	1	Identify the report as a systematic review.	X
**ABSTRACT**			
Abstract	2	See the PRISMA 2020 for Abstracts checklist.	X
**INTRODUCTION**			
Rationale	3	Describe the rationale for the review in the context of existing knowledge.	X
Objectives	4	Provide an explicit statement of the objective(s) or question(s) the review addresses.	X
**METHODS**			
Eligibility criteria	5	Specify the inclusion and exclusion criteria for the review and how studies were grouped for the syntheses.	X
Information sources	6	Specify all databases, registers, websites, organisations, reference lists and other sources searched or consulted to identify studies. Specify the date when each source was last searched or consulted.	X
Search strategy	7	Present the full search strategies for all databases, registers and websites, including any filters and limits used.	X
Selection process	8	Specify the methods used to decide whether a study met the inclusion criteria of the review, including how many reviewers screened each record and each report retrieved, whether they worked independently, and if applicable, details of automation tools used in the process.	X
Data collection process	9	Specify the methods used to collect data from reports, including how many reviewers collected data from each report, whether they worked independently, any processes for obtaining or confirming data from study investigators, and if applicable, details of automation tools used in the process.	X
Data items	10a	List and define all outcomes for which data were sought. Specify whether all results that were compatible with each outcome domain in each study were sought (e.g. for all measures, time points, analyses), and if not, the methods used to decide which results to collect.	X
	10b	List and define all other variables for which data were sought (e.g. participant and intervention characteristics, funding sources). Describe any assumptions made about any missing or unclear information.	X
Study risk of bias assessment	11	Specify the methods used to assess risk of bias in the included studies, including details of the tool(s) used, how many reviewers assessed each study and whether they worked independently, and if applicable, details of automation tools used in the process.	X
Effect measures	12	Specify for each outcome the effect measure(s) (e.g. risk ratio, mean difference) used in the synthesis or presentation of results.	X
Synthesis methods	13a	Describe the processes used to decide which studies were eligible for each synthesis (e.g. tabulating the study intervention characteristics and comparing against the planned groups for each synthesis (item #5)).	X
	13b	Describe any methods required to prepare the data for presentation or synthesis, such as handling of missing summary statistics, or data	X

Section and Topic	Item #	Checklist item	Location where item is reported
		conversions.	
	13c	Describe any methods used to tabulate or visually display results of individual studies and syntheses.	**X**
	13d	Describe any methods used to synthesize results and provide a rationale for the choice(s). If meta-analysis was performed, describe the model(s), method(s) to identify the presence and extent of statistical heterogeneity, and software package(s) used.	**X**
	13e	Describe any methods used to explore possible causes of heterogeneity among study results (e.g. subgroup analysis, meta-regression).	**X**
	13f	Describe any sensitivity analyses conducted to assess robustness of the synthesized results.	**X**
Reporting bias assessment	14	Describe any methods used to assess risk of bias due to missing results in a synthesis (arising from reporting biases).	**X**
Certainty assessment	15	Describe any methods used to assess certainty (or confidence) in the body of evidence for an outcome.	**X**
**RESULTS**			
Study selection	16a	Describe the results of the search and selection process, from the number of records identified in the search to the number of studies included in the review, ideally using a flow diagram.	**X**
	16b	Cite studies that might appear to meet the inclusion criteria, but which were excluded, and explain why they were excluded.	**X**
Study characteristics	17	Cite each included study and present its characteristics.	**X**
Risk of bias in studies	18	Present assessments of risk of bias for each included study.	**X**
Results of individual studies	19	For all outcomes, present, for each study: (a) summary statistics for each group (where appropriate) and (b) an effect estimate and its precision (e.g. confidence/credible interval), ideally using structured tables or plots.	**X**
Results of syntheses	20a	For each synthesis, briefly summarise the characteristics and risk of bias among contributing studies.	**X**
	20b	Present results of all statistical syntheses conducted. If meta-analysis was done, present for each the summary estimate and its precision (e.g. confidence/credible interval) and measures of statistical heterogeneity. If comparing groups, describe the direction of the effect.	**X**
	20c	Present results of all investigations of possible causes of heterogeneity among study results.	**X**
	20d	Present results of all sensitivity analyses conducted to assess the robustness of the synthesized results.	**X**
Reporting biases	21	Present assessments of risk of bias due to missing results (arising from reporting biases) for each synthesis assessed.	**X**
Certainty of evidence	22	Present assessments of certainty (or confidence) in the body of evidence for each outcome assessed.	**X**
**DISCUSSION**			

Section and Topic	Item #	Checklist item	Location where item is reported
Discussion	23a	Provide a general interpretation of the results in the context of other evidence.	**X**
	23b	Discuss any limitations of the evidence included in the review.	**X**
	23c	Discuss any limitations of the review processes used.	**X**
	23d	Discuss implications of the results for practice, policy, and future research.	**X**
**OTHER INFORMATION**			
Registration and protocol	24a	Provide registration information for the review, including register name and registration number, or state that the review was not registered.	**X**
	24b	Indicate where the review protocol can be accessed, or state that a protocol was not prepared.	**X**
	24c	Describe and explain any amendments to information provided at registration or in the protocol.	**X**
Support	25	Describe sources of financial or non-financial support for the review, and the role of the funders or sponsors in the review.	**X**
Competing interests	26	Declare any competing interests of review authors.	**X**
Availability of data, code and other materials	27	Report which of the following are publicly available and where they can be found: template data collection forms; data extracted from included studies; data used for all analyses; analytic code; any other materials used in the review.	**X**

From: Page MJ, McKenzie JE, Bossuyt PM, Boutron I, Hoffmann TC, Mulrow CD, et al. The PRISMA 2020 statement: an updated guideline for reporting systematic reviews. BMJ 2021;372:n71. doi: 10.1136/bmj.n71
